# Axon guidance gene-targeted siRNA delivery system improves neural stem cell transplantation therapy after spinal cord injury

**DOI:** 10.1186/s40824-023-00434-2

**Published:** 2023-10-15

**Authors:** Seong Jun Kim, Wan-Kyu Ko, Gong Ho Han, Daye Lee, Min Jai Cho, Seung Hun Sheen, Seil Sohn

**Affiliations:** 1 Department of Neurosurgery, CHA Bundang Medical Center, CHA University, 59, Yatap-ro, Bundang- gu, Seongnam-si, 13496 Gyeonggi-do Republic of Korea; 2 Department of Biomedical Science, CHA University, 335, Pangyo-ro, Bundang-gu, Seongnam-si, 13488 Gyeonggi-do Republic of Korea; 3 Department of Neurosurgery, Chungbuk National University, 776, 1Sunhawn-ro, Seowon-gu, Cheongju-si, 28644 Republic of Korea

**Keywords:** Spinal cord injuries, Neural stem cells, Semaphorin-3A, Small interfering RNA, Axon guidance

## Abstract

**Background:**

Neural stem cells (NSCs) derived from the embryonic spinal cord are excellent candidates for the cellular regeneration of lost neural cells after spinal cord injury (SCI). Semaphorin 3 A (Sema3A) is well known as being implicated in the major axon guidance of the growth cone as a repulsive function during the development of the central nervous system, yet its function in NSC transplantation therapy for SCI has not been investigated. Here, we report for the first time that embryonic spinal cord-derived NSCs significantly express Sema3A in the SCI environment, potentially facilitating inhibition of cell proliferation after transplantation.

**Methods:**

siRNA-Sema3A was conjugated with poly-l-lysin-coated gold nanoparticles (AuNPs) through a charge interaction process. NSCs were isolated from embryonic spinal cords of rats. Then, the cells were embedded into a dual-degradable hydrogel with the siRNA- Sema3A loaded-AuNPs and transplanted after complete SCI in rats.

**Results:**

The knockdown of Sema3A by delivering siRNA nanoparticles via dual-degradable hydrogels led to a significant increase in cell survival and neuronal differentiation of the transplanted NSCs after SCI. Of note, the knockdown of Sema3A increased the synaptic connectivity of transplanted NSC in the injured spinal cord. Moreover, extracellular matrix molecule and functional recovery were significantly improved in Sema3A-inhibited rats compared to those in rats with only NSCs transplanted.

**Conclusions:**

These findings demonstrate the important role of Sema3A in NSC transplantation therapy, which may be considered as a future cell transplantation therapy for SCI cases.

**Graphical Abstract:**

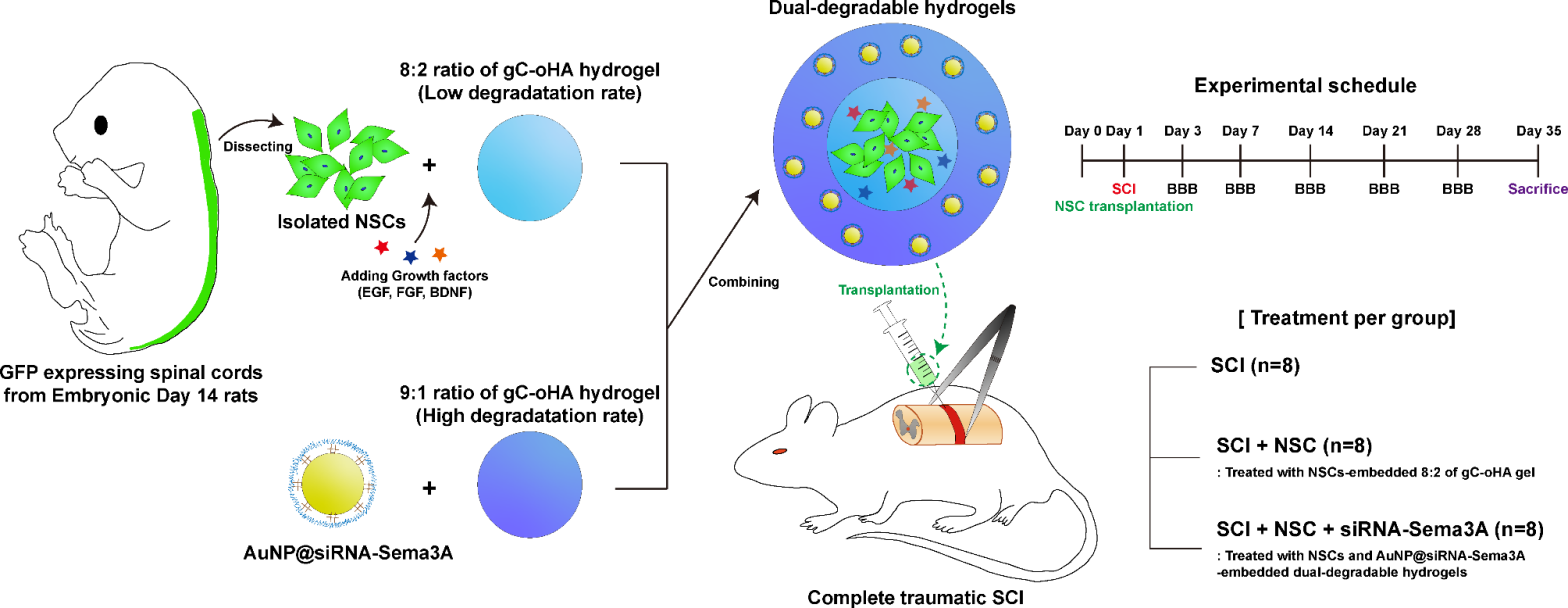

**Supplementary Information:**

The online version contains supplementary material available at 10.1186/s40824-023-00434-2.

## Introduction

Spinal cord injury (SCI) is a devastating traumatic condition that requires regenerative treatment interventions because the central nervous system (CNS) lacks the ability to recover spontaneously. Neural stem cell (NSC) transplantation has the potential to be an effective treatment modality because stem cells can replace lost neural cells in an injured spinal cord [1–3]. Specifically, NSCs from the spinal cords of embryos are promising candidates for cell therapeutics because NSCs are pluripotent and can thus differentiate into neurons, astrocytes, and oligodendrocytes [4–6]. However, there remain several challenges that restrict NSC transplantation as a clinical application, including the impairment of functional recovery due to non-specific synapse formation, poor regenerative capability, and the low survival of transplanted NSCs in the injury milieu [7–9].

Semaphorin-3 A (Sema3A) is a well-known repulsive guidance protein that functions during the signaling growth cone collapse process in the nervous system [10–12]. Sema3A is involved in many developmental processes that shape the CNS, including neuronal proliferation, migration, neuritogenesis, and synapse formation by restricting axonal outgrowth during embryonic neurogenesis [13–16]. Although the differentiation procedure of embryonic-derived NSC recapitulates the developmental processes of the CNS [17], to the best of our knowledge, the role of Sema3A in transplanted NSCs after SCI has not been explored. Given that the differentiating process of NSCs derived from the fetal spinal cord coincides with embryonic CNS development, we hypothesized that Sema3A disturbs the integration with grafted NSCs and the host cells after NSC transplantation following SCI.

## Materials and methods

### Preparation and characterization of Sema3A siRNA-loaded gold nanoparticles (AuNP@siRNA-Sema3A)

We synthesized gold nanoparticles (AuNPs) as described previously [18, 19]. Briefly, a 200 mL two-necked flask and a magnetic stir bar were soaked in aqua regia for at least 15 min. Next, 98 mL of deionized water (DW) was loaded into the two-necked flask. An amount of 2 mL of a 50 mM chloroauric acid solution (Sigma Aldrich, St. Louis, MO, USA) was added to a final concentration of 1 mM. When the solution begins to reflux, 10 mL of 38.8 mM sodium citrate (Sigma Aldrich, St. Louis, MO, USA) was quickly added. After 15 min, the color of the solution changed to deep red, indicating the 15 nm AuNPs existed in the solution.

Sema3A siRNA (siRNA-Sema3A) was obtained from Bioneer (Seoul, South Korea). The synthesized AuNPs (7.36 nM) were diluted with DW to concentrations of 1 nM. To conjugate siRNA-Sema3A onto the AuNPs, a protocol established by Lee et al. was used [20]. The sequences of siRNA-Sema3A used here were as follows: sense strand: 5′-GAGGAACGGAGUAGACUG-3′ and antisense strand: 5′-ACAGUCUACUCCGUUCCU-3′. A poly-l-lysine (PLL) solution (Molecular weight: 22.5 kDa; Sigma Aldrich) was prepared by dissolving PLL powder in DW (5 mg/ mL). Then, 1 mL of a 1 nM AuNP solution was added dropwise into 0.5 mL of PLL solution. After incubating for 30 min in the dark with gentle shaking, the solution was centrifuged for 15 min at 16,100 × g. The supernatant was removed and resuspended with DW and was then centrifuged for 15 min at 16,100 × g. After one more wash, the PLL-coated AuNPs were resuspended in 0.5 mL of DW, and the siRNA solution (2 nM, 0.25 mL) was added dropwise to all of the PLL-coated solution. The reaction solution was incubated in the dark for 30 min with gentle shaking, followed by three washes with DW. The final concentrations of AuNP@siRNA-Sema3A used for the subsequent in vitro and in vivo experiments were 1 nM. The synthesized AuNPs, PLL-coated AuNPs, and AuNP@siRNA-Sema3A were measured to determine the surface charges using the zeta potential (Zetasizer 2000, Malvern, Worcestershire, UK) and were observed as well through transmission electron microscopy (H-7100, Hitachi, Japan). The coating ratio between the AuNPs and siRNA-Sema3A was analyzed by means of a SYBR gold assay [21].

### Dissociation and isolation of rat embryonic day 14 (E14)-derived NSCs

E14 spinal cords expressing green fluorescent protein (GFP) under the ubiquitin C promoter were provided from transgenic Fischer 344-Tg (EGFP) rats (n = 14; Rat Resource and Research Center, Columbia, MO).The E14 spinal cords were dissected and dissociated as described in the literature [22, 23]. Briefly, the embryos were dissected at days 13.5–14.5 after identifying the vaginal plug insertion, where the spinal cords of GFP-positive fetuses were dissected. Dissociated spinal cords were digested in 0.25% trypsin-ethylenediaminetetraacetic acid for ten minutes at 37 °C, centrifuged, and counted using a hemocytometer. E14-derived NSCs were cultured using Neurobasal™ plus medium (GIBCO, Carlsbad, CA, USA) containing the B27 supplement and 1% penicillin–streptomycin.

### Assessing the cellular uptake of AuNP@siRNA-Sema3A using immunocytochemistry

NSCs were cultured in glass-bottomed dishes and respectively treated with AuNP, Hydrogel, and AuNP@siRNA-Sema3A. At predetermined time points, the cells were washed and fixed with 4% paraformaldehyde. Subsequently, immunocytochemistry staining was conducted as previously described [24]. The primary antibodies were as follows: rabbit anti-Tuj1 (1:200, Abcam, #ab221935) and mouse anti-Sema3A (1:200, Santacruz, #sc-74,554). Fluorescence secondary antibodies were conjugated to Alexa 568 (1:1000, Molecular Probes, #A11037) or Alexa 647 (1:1000, Molecular Probes, #A21236). Nuclei were stained with 4′,6′-diamidino-2-phenylindole dihydrochloride (DAPI; 2 ng/ml, Molecular Probes). The cyanine 5.5 dye (Cy5.5)-labeled AuNP@siRNA-Sema3A and the expression of immunocytochemistry staining were detected using a confocal laser-scanning microscope (LSM 880, Carl Zeiss, Jena, Germany). Sema3A expression outcomes were determined from three designated regions of interest (ROI; 127.97 μm × 127.97 μm) in composite tiled scan images (436.82 μm × 436.82 μm) for each group. Cell numbers were quantified using DAPI, where the treated groups were normalized to the non-treated controls (normalized to a 1-fold change).

### Axon outgrowth assay

NSCs were seeded onto a poly-d-lysine-coated SPL Scar™ Block (2 × 10^4^ / well; SPL Life Sciences, Pocheon, South Korea) to allow cell attachment inside one compartment of the block. One day after seeding, the block was removed from the plate and the culture medium was treated in each case with AuNP, Hydrogel, and AuNP@siRNA-Sema3A. After seven days, axons were stained with Tuj1, and the length of the axons was measured over the blocked-off area. ROIs (196 × 196 μm, n = 5 per group) across areas of the Tuj1 were quantified using ImageJ software (NIH).

### Hydrogels formation and characterization

The glycol chitosan (gC; MW: 50 kDa) used here was purchased from Sigma Aldrich (St. Louis, MO, USA). Oxidized hyaluronic acid (oHA) was prepared as previously described [25, 26]. Lyophilized oHA and gC powder were separately dissolved in saline (2% gC and 3% oHA), after which the 2% gC and 3% oHA solutions were then mixed at ratios of 8:2 and 9:1, respectively. The storage elastic modulus (G′) and loss modulus (G″) of each synthesized hydrogels were measured using a rotating rheometer (MCR-92; Anton paar, Graz, Austria). The temperature was held at 25 °C during the viscoelastic measurements, and the frequency was varied from 0.1 to 1 Hz. The strain of the samples was kept at 0.1 N.

### Assessing cell viability in hydrogels

NSCs were seeded into the 2% gC solution and mixed with the 3% oHA solution for gel formation (2 × 10^5^ cells per each gel). These samples were then placed on 48-well culture plates and were incubated with a culture medium for 24 h at 37℃ in 5% CO_2_. The cell supernatants in each gel (n = 3 per group) were measured using a cell viability assay kit (EZ-Cytox, Daeil Lab Service, Seoul, Korea) and quantified using UV-visible spectroscopy. The absorbance levels of the NSCs seeded in the culture dishes were normalized as 100% viable. Next, the gels containing cells were washed and incubated in Alexa Fluor 488 phalloidin (Invitrogen, Waltham, MA, USA) for 30 min at room temperature. The NSCs embedded into the gel were visualized using a confocal laser-scanning microscope with z-stack imaging.

### Animals

Adult female Sprague Dawley rats (190–230 g, n = 26) were handled in accordance with the regulations of the Institutional Animal Care and Use Committee (IACUC) of CHA University (IACUC200217) and according to the Guide for the Care and Use of Laboratory Animals (NIH; Bethesda, MD, USA). SCI surgeries were done under deep anesthesia induced by a combination of tiletamine-zolazepam (50 mg/ kg) and xylazine (10 mg/ kg). After surgery, the rats were kept warm and housed separately, with *ad libitum* access to food. Manual bladder massage was conducted daily to assist with urination.

### Hydrogel degradation test in vivo

The previously prepared 2% gC solution was added to Cy5.5 and mixed with 3% oHA at 8:2 and 9:1 ratios, respectively. Under anesthesia with isoflurane, the Cy5.5-labeled hydrogel gels were transplanted subcutaneously under the middle dorsal regions of the skin (Three sites per rats). At indicated timepoints, the expressions of Cy5.5 in the transplanted hydrogel were recorded using a Pearl Impulse small animal imaging system (LI-COR Biosciences, Lincoln, NE). The expression of each gel on day 1 was quantified as 100% and relative values were then measured.

### Preparation of hydrogels including NSCs and AuNP@siRNA-Sema3A

To create single-layer hydrogels, NSCs were resuspended at a concentration of 2 × 10^5^ cells/ µL in the 2% gC and 3% oHA solutions containing the following growth factors: Brain-derived neurotrophic factor (50 mg/ml, Peprotech), vascular epidermal growth factor (VEGF; 10 mg/ml, Peprotech), and fibroblast growth factor (10 mg/ml, Peprotech). The 2% gC and 3% oHA solution containing cells were mixed at an 8:2 ratio.

To create double-layer hydrogels of siRNA-Sema3A and NSCs, 8:2 gels containing NSCs at a concentration of 400,000 cells/µL were prepared as described above and were mixed at an 8:2 ratio. The 8:2 gels were chopped into small pieces on a petri dish.

The prepared AuNP@siRNA-Sema3A samples were resuspended at a concentration of 0.1 nM in a new 2% gC and 3% oHA solutions, respectively. The 2% gC and 3% oHA solutions containing AuNP@siRNA-Sema3A were mixed at a 9:1 ratio on the chopped 8:2 gels.

### Traumatic SCI modeling and transplantation surgeries

For the SCI, after the rats were anesthetized, a midline incision was made in the lower back. Tissues were dissected layer by layer to reveal the T8-T10 vertebra. A T9 total laminectomy was conducted to expose the dura. We induced a severe SCI at the T9 laminectomy site by completely compressing the entire spinal cord laterally using Dumont #2 forceps (#11223-20; Fine Science Tools, CA, USA).

After the injury, the rats were immediately injected with saline as a control (SCI group, n = 8), single-layer hydrogels embedding NSCs (SCI + NSC group, n = 8), or double-layer hydrogels including NSCs and AuNP@siRNA-Sema3A (SCI + NSC + AuNP@siRNA-Sema3A group, n = 8) using a stereotactic microinjector. Each group was injected at three sites (1.2 × 10^6^ cells per sites): each injection at the rostral and caudal sites from the lesion epicenter and one injection at the lesion epicenter, spaced 1 mm apart. Injections were applied 1.5 mm below the surface at 0.1 uL/ second for 30 s. All rats received an analgesic (Carprofen, Pfizer, Italy, 5 mg/ kg) and an antibiotic (Baytril, Bayer Health Care AG, 5 mg/ kg) daily for three days after surgery.

### Hindlimb locomotor evaluation

The Basso, Beattie, and Bresnahan (BBB) scale is a 22-point scale in which 0 indicates no observed hindlimb movements and 21 is representative of a normal ambulating rodent [27]. At 1, 3, 5, 7, 14, 21, 28, and 35 days after SCI, hindlimb movements were evaluated by two investigators blind to the experimental conditions using BBB scoring.

### Immunohistochemistry

After terminal anesthesia by overdose, rats were perfused transcardially with 4% paraformaldehyde. The spinal cords were then collected, embedded in paraffin, and were processed for immunofluorescence as previously described [28]. The primary antibodies were as follows: rabbit anti-glial fibrillary acidic protein (GFAP; 1:200, Abcam, #ab7260), mouse anti-GFAP (1:200, Thermo fisher, #MA5-12023), mouse anti-Sema3A (1:200, Santa cruz, #sc-74,544), rabbit anti-Tuj1 (1:200, Abcam, #ab221935), guineapig anti-Homer (1:200, Synaptic systems, #160 004), and rabbit anti-laminin (1:200, Abcam, #PA1-16730). Fluorescence secondary antibodies were conjugated to Alexa 488 (1:1,000, Thermo fisher), Alexa 594 (1:1,000, Thermo fisher), or Alexa 647 (1:1,000, Thermo fisher, #21,236). Nuclei were stained with DAPI (2 ng/ml, Invitrogen, D1306). The sections were mounted using a fluorescence mounting medium (DAKO, Glostrup, Denmark). All sections were examined and imaged using confocal laser scanning microscopy.

### Quantification of immunohistochemistry

The Sema3A intensity levels were determined by designating three ROIs (415.13 μm × 415.13 μm) within the lesion center or around the glia scar in composite tiled scan images (2,380 μm × 2,550 μm). Post-synaptic activity of transplanted cells was determined in each case using Homer by designating three ROIs (212.55 μm × 212.55 μm) within the graft in composite tiled scan images (3,250 μm × 2,850 μm). The calculated immunofluorescence intensities were quantified automatically using the ‘arithmetic mean intensity’ method in Zen Blue (v.3.2) software. NSC survival rates were determined by counting GFP-labeled cells within a fixed box 332.11 μm × 332.11 μm in size within the graft. The intensity of the GFP-labeled cells in the SCI + NSC group was set to 1-fold, with the intensity of the GFP-labeled cells in the SCI + NSC + siRNA-Sema3A group then relatively quantified using ImageJ software. Sections stained for laminin were scanned using constant exposure settings. Single-channel immunofluorescence images were converted to black and white, and the stained area per total area in the ROI was automatically measured by thresholding in the ImageJ software. The same threshold was applied to all analyzed images within each experiment.

### Statistical analyses

Multiple comparisons among the three groups were conducted with a one-way analysis of variance (ANOVA), and Tukey’s multiple-comparison test was used as a post-hoc analysis method. Two-group comparisons were conducted with Student’s t-tests. Differences in p-values for which ^*^p < 0.05, ^**^p < 0.01, and ^***^p < 0.001 were deemed statistically significant. All statistical analyses and graphing of data were performed using GraphPad Prism.

## Results

### Characterization of AuNP@siRNA-Sema3A

To investigate the role of Sema3A in embryonic-derived NSCs, we synthesized AuNPs for the delivery of siRNA-Sema3A.

**Fig. 1:**
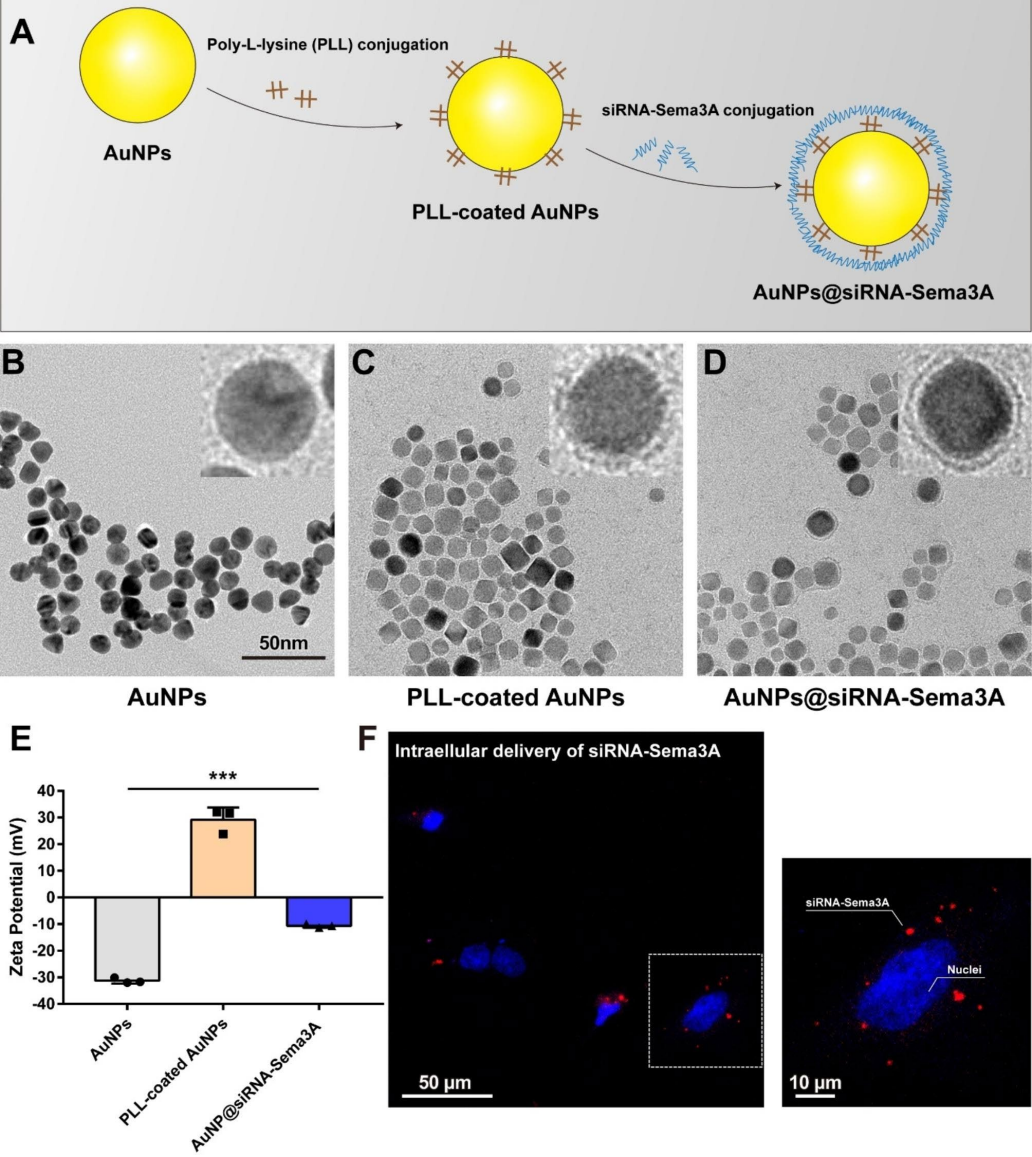
**(A)** Schematic illustration of the synthesis of AuNP@siRNA-Sema3A using electrostatic interactions. TEM images of **(B)** AuNP, **(C)** AuNP-PLL, and **(D)** AuNP@siRNA-Sema3A. **(E)** Changes in the surface charge of each AuNP according to the step-by-step coating of PLL and siRNA-Sema3A. **(F)** Confocal micrograph of NSCs treated with Cy5.5-labeled AuNP@siRNA-Sema3A (left) and details (right) of the internalization of Cy5.5-labeled AuNP@siRNA-Sema3A into the cells

AuNPs were coated with PLL, a positively charged polyelectrolyte. siRNA-Sema3A, being negatively charged, was conjugated to the PLL-coated AuNPs through charge interaction (Fig. 1A). PLL-coated AuNPs and AuNP@siRNA-Sema3A showed a ~ 2–3 nm increase in the particle diameter compared with bare AuNPs (Fig. 1B-D). The initial surface charge of the bare AuNPs was confirmed by ensuring that the zeta potential was − 40 mV. The PLL coating increased the surface charge to approximately + 46 mV, while the subsequent siRNA layer decreased surface charge to about − 30 mV (Fig. 1E) again. This indicates that siRNA-Sema3A was successfully loaded onto the surfaces of the AuNPs. The coating ratio of siRNA-Sema3A with AuNPs is approximately 92.42%, where 93.42 is the percentage value obtained from the SYBR gold assay (Fig. S1). To confirm the intracellular delivery efficiency of AuNP@siRNA-Sema3A, E14-derived NSCs were dissociated, cultured, and treated with the Cy5.5-labeled AuNP@siRNA-Sema3A. Figure 1 F shows strong fluorescence around the nuclei, demonstrating the effective cellular internalization of AuNP@siRNA-Sema3A.

### Sema3A is expressed by NSCs and regulates axonal growth

To evaluate whether Sema3A is produced during NSC differentiation, we carried out an immunocytochemistry assessment of Sema3A and Tuj1, a marker of neurons, in the cultured E14-NSCs. Subsequently, we conducted treatments with AuNPs, Hydrogels, and AuNP@siRNA-Sema3A lasting three days in each case.

**Fig. 2:**
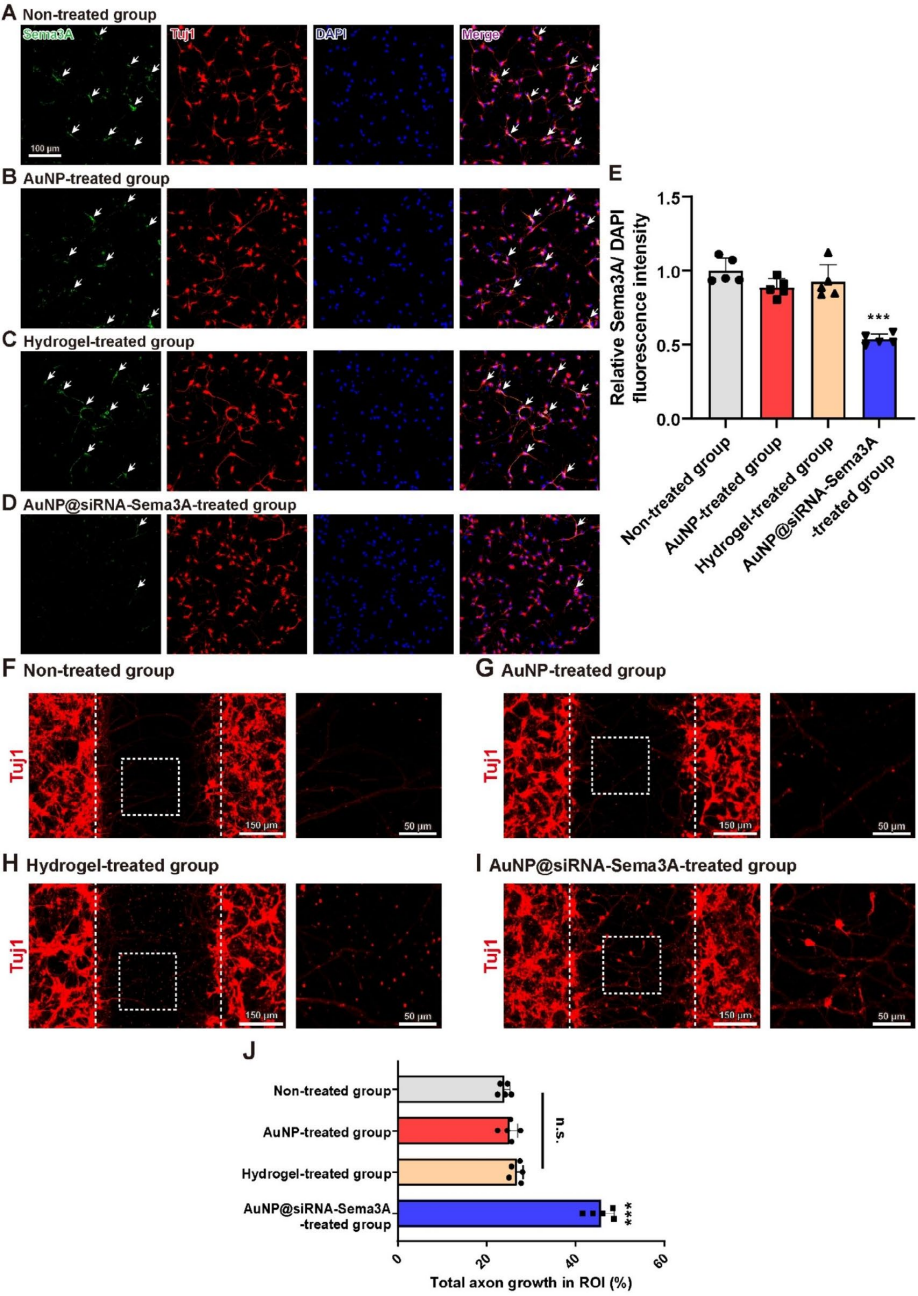
Differentiating NSCs express Sema3A, and AuNP@siRNA-Sema3A suppresses Sema3A expression **(A-D)** Representative immunofluorescence images of Sema3A (anti-Sema3A, Green), neurons (anti-Tuj1, Red), and nuclei (DAPI, Blue) of the **(A)** non-treated, **(B)** AuNP, **(C)** Hydrogel, and **(D)** AuNP@siRNA-Sema3A groups. **(E)** Quantitative analyses of the fluorescence intensity for Sema3A. **(F-I)** Representative immunofluorescence images for Tuj1-labelled axon growth of the **(F)** non-treated group, **(G)** AuNP, **(H)** Hydrogel, and **(I)** AuNP@siRNA-Sema3A groups. **(J)** Quantitative analyses of Tuj1-labelled total axon growth in ROIs. Results are the mean ± standard error of the mean (SEM); ^***^p < 0.001. one-way ANOVA with Tukey post-hoc test

In the cultured E14-NSCs without any treatment, Sema3A was colocalized in the form of Sema3A and Tuj1 markers (Fig. 2A). Upon a comparison with the non-treated group, there were no significant differences in the Sema3A expression levels within both carrier groups (Fig. 2B, C, E; Non-treated group: 1.00 ± 0.08, AuNP group: 0.88 ± 0.06, Hydrogel group: 0.92 ± 0.12). However, Sema3A in the AuNP@siRNA-Sema3A group was significantly decreased compared to that in the non-treated group (Fig. 2D, E; AuNP@siRNA-Sema3A group: 0.53 ± 0.04, ^***^p < 0.001). These results indicate that Sema3A is in fact expressed in differentiating NSCs and significantly suppressed using AuNP@siRNA-Sema3A.

To assess the morphological changes of NSCs with each treatment, axonal growth was measured for ten days. Total axonal growth in the ROIs of the non-treated group was detected at an average level of 24.00 ± 1.25 (Fig. 2F, J). Upon a comparison with the non-treated group, there were no significant differences in the total axonal growth within both carrier groups (Fig. 2G, H, J; AuNP group: 25.15 ± 1.86, Hydrogel group: 26.80 ± 1.37). However, total axonal growth in the ROIs of the AuNP@siRNA-Sema3A group was significantly increased compared to that in the ROI of the non-treated group (Fig. 2I, J; AuNP@siRNA-Sema3A group: 45.76 ± 3.00, ^***^p < 0.001).

### Preparation of degradable hydrogels for delivering the siRNA of Sema3A

Control over the AuNP@siRNA-Sema3A release and the simultaneous promotion of the survival of grafted NSCs within the injury microenvironment are crucial for the development of an optimal NSC transplant. However, a single hydrogel cannot simultaneously perform the functions of short-term drug release (~ 2 weeks) and long-term grafted cell retention (~ 5 weeks). Thus, a multifunctional dual-degradable hydrogel was designed for this purpose. The dual-degradable hydrogels samples were synthesized using gC and oHA at corresponding ratios of 8:2 and 9:1.

**Fig. 3:**
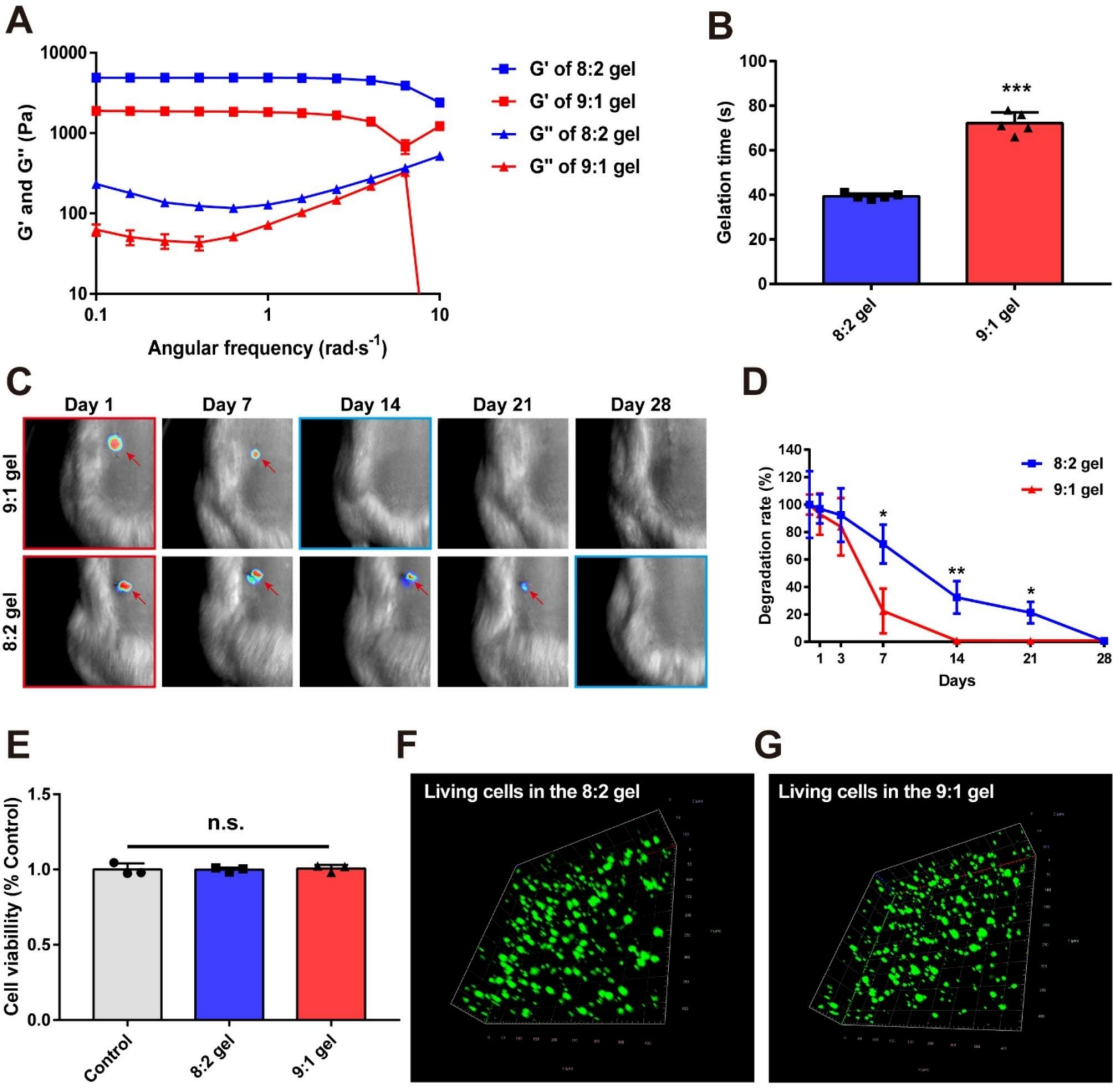
**(A)** Characterization of the rheological properties of the 8:2 and 9:1 gels. **(B)** Quantitative analyses of the gelation time for the 8:2 gel and 9:1 gels **(C)** Representative bioluminescence image after the transplant of Cy5.5-labeled 8:2 gel or 9:1 gel and **(D)** quantitative analyses of the gel degradation rate. Results are the mean ± standard deviation (SD); ^*^p < 0.05 and ^**^p < 0.01. Unpaired two-tailed Student’s t-tests. **(E)** Quantitative analyses of the cell viability in each hydrogel. Results are the mean ± SEM; n.s. is not significant. one-way ANOVA with Tukey post-hoc test. **(F, G)** Immunofluorescence analysis of GFP-labeled NSCs in **(F)** the 8:2 and **(G)** the 9:1 gel

G′ of the 8:2 gel was higher than that of the 9:1 gel (Fig. 3A). Moreover, G′′ of the 8:2 gel was higher than that of the 9:1 gel. The time during which the hydrogel is structured was also shorter in the 8:2 gel than in the 9:1 gel, indicating that oHA was more assembled in the cross-linking 8:2 gel than in the 9:1 gel (Fig. 3B). To verify the degradation rates of the synthesized hydrogels due to cross-linking, we quantified the degradation rates of the 8:2 gel and the 9:1 gel in vivo by loading each hydrogel with fluorescent Cy5.5. The fluorescence of Cy5.5 in the 8:2 gel gradually decreased, reaching a state of complete degradation within 14 days, whereas the fluorescence of Cy5.5 in the 8:2 gel was still observed at this time (Figs. 3C and D and 32.36 ± 11.78%, ^**^p < 0.01). The 8:2 gel was completely degraded within 28 days.

A cell viability test was conducted to ensure that the 8:2 and 9:1 gels do not cause any unwanted cellular toxicity. The cell viability of the 8:2 gel and the 9:1 gel group did not produce any significant differences relative to the control group (Fig. 3E). Moreover, no differences in the cell densities were found in the three-dimensional GFP-labeled NSC images (Fig. 3F, G), suggesting that our hydrogels do not affect the proliferation of NSCs. Taken together, our designed dual-degradable hydrogels can release AuNP@siRNA-Sema3A for a short-term subacute SCI while simultaneously providing a scaffold for a long-term cell growth environment.

### Grafted NSCs express Sema3A after SCI

To evaluate the extent of the AuNP@siRNA-Sema3A treatment in the transplanted NSCs after SCI, NSCs were dissected from E14 spinal cords from pregnant transgenic rats that ubiquitously express GFP. The cells were embedded into the dual-degradable gel with siRNA-Sema3A and transplanted after complete SCI in the rats.

**Fig. 4:**
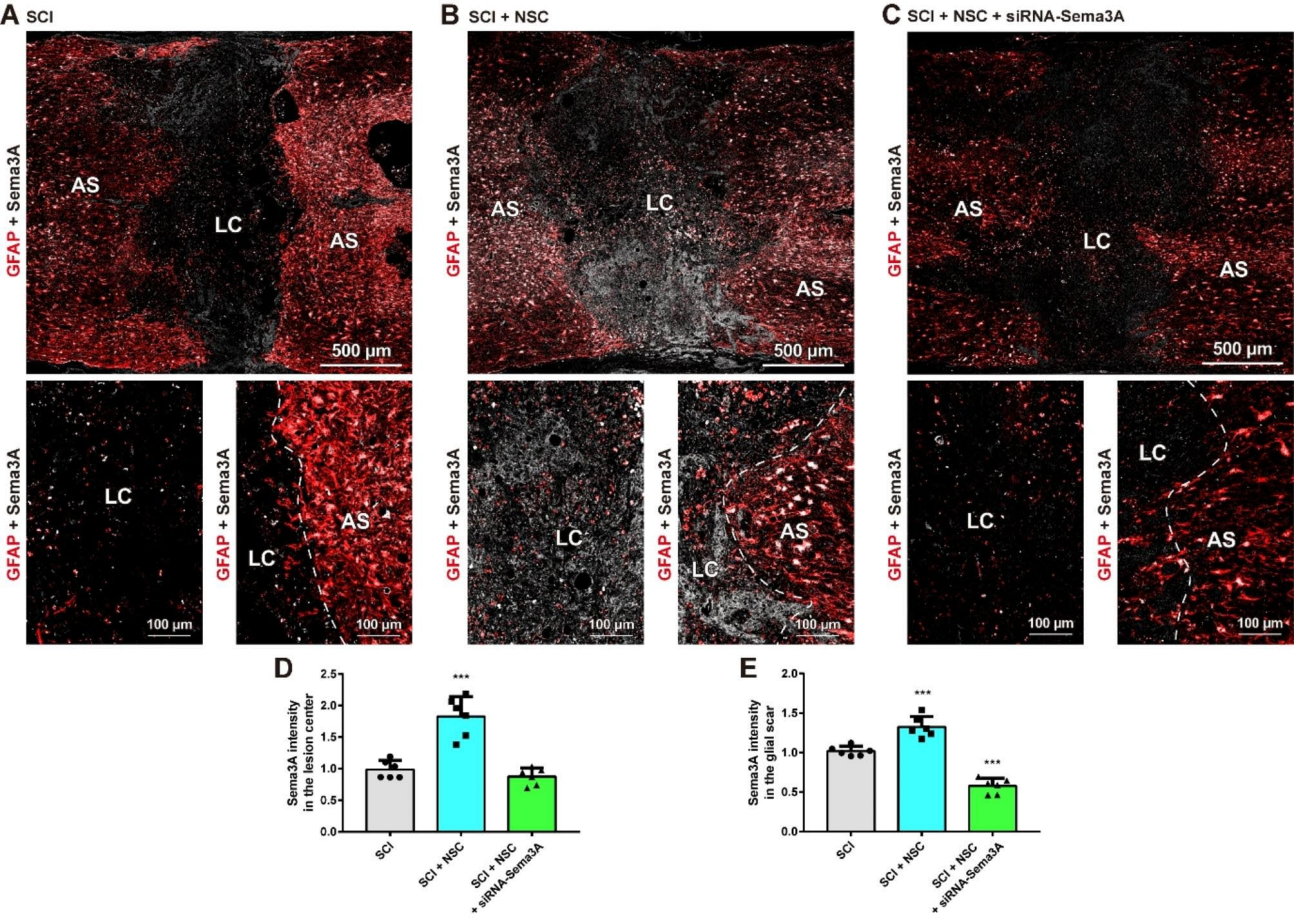
Transplantation of NSCs induces Sema3A in the grafted site and AuNP@siRNA-Sema3A successfully inhibits the expression of Sema3A after SCI. Immunofluorescence analysis in composite tiled scans of transverse sections stained for glial scarring (anti-GFAP, Red) and Sema3A (anti-Sema3A, white). Astrocytic glial scar (AS) around the lesion center (LC). **(A)** Representative merged image of GFAP and Sema3A in the SCI group (top). Higher magnification in the LC (left) and in the AS (right) of the SCI group. **(B)** Representative merged image of GFAP and Sema3A in the SCI + NSC group (top). Higher magnification in the LC (left) and in the AS (right) of the SCI + NSC group. **(C)** Representative merged image of GFAP and Sema3A in the SCI + NSC + siRNA-Sema3A group (top). Higher magnification in the LC (left) and in the AS (right) of the SCI + NSC + siRNA-Sema3A group. **(D-E)** Quantitative analyses of the fluorescence intensity for Sema3A in the **(D)** LC and **(E)** AS. Results are the mean ± SEM; ^***^p < 0.001. one-way ANOVA with Tukey post hoc test

Five weeks after injury, Sema3A was observed around the GFAP-positive glial scar, opposed to the lesion center (Fig. 4A, D; SCI group: 0.99 ± 0.14). However, the amount of Sema3A in NSC-only transplanted rats increased significantly in the lesion center (Fig. 4B, D; SCI + NSC group: 1.83 ± 0.31, ^***^p < 0.001), indicating that grafted NSCs induces the expression of Sema3A after transplantation. On the other hand, Sema3A expression in the siRNA-Sema3A-treated rats was significantly decreased in the lesion center compared to that in the NSC transplanted rats (Fig. 4C, D; SCI + siRNA-Sema3A + NSC group: 0.87 ± 0.13, ^***^p < 0.001). This tendency was also noted at the glial scar site. Sema3A in the NSC-only transplanted rats was significantly increased around the GFAP-positive glial scar sites compared to that in the SCI rats (Fig. 4A, B, E; SCI group: 1.02 ± 0.06, SCI + NSC group: 1.32 ± 0.13, ^***^p < 0.001), whereas Sema3A in the siRNA-Sema3A-treated rats was significantly decreased around the GFAP-positive glial scar sites compared to that in the NSC-only transplanted rats (Fig. 4C, E; SCI + siRNA-Sema3A + NSC group: 0.58 ± 0.10, ^***^p < 0.001). In addition, Sema3A in the siRNA-Sema3A-treated rats was significantly decreased at the glial scars compared to that in the SCI group (^***^p < 0.001). These findings show that NSC transplantation induces Sema3A in the grafted site, which localizes around the glial scar after SCI. Most notably, we confirmed that siRNA-Sema3A loaded on AuNP can successfully inhibit the expression of Sema3A.

### Knockdown of Sema3A enhanced transplanted NSC survival after SCI

To investigate whether the knockdown of Sema3A influenced cell survival and localization of transplanted NSCs after SCI, immunofluorescence assessments of GFP-labeled NSCs from GFAP + glial scars were conducted.

**Fig. 5:**
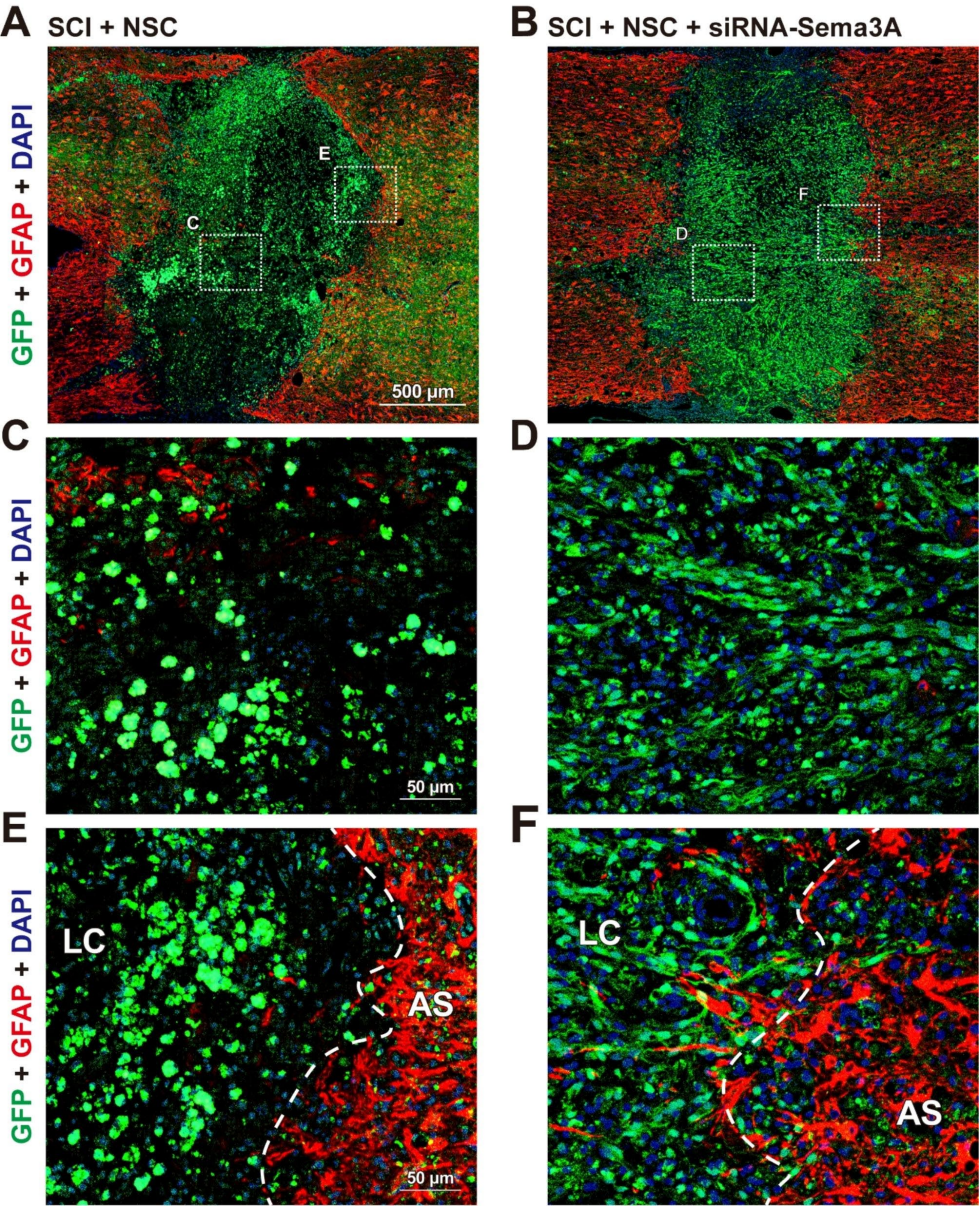
Knockdown of Sema3A increases survival of grafted NSCs and induces neural differentiation after transplantation in the injured spinal cord. Immunofluorescence analysis in composite tiled scans of transverse sections stained for GFP-labeled NSCs (anti-GFP, Green) and glial scar (anti-GFAP, Red). **(A, B)** Representative merged image for GFP and GFAP in the **(A)** SCI + NSC group, and **(B)** SCI + NSC + siRNA-Sema3A group. **(C, D)** Higher magnification in the LC (boxed area) **(C)** from A and **(D)** from B. **(E, F)** Higher magnification of the AS (boxed area) **(E)** from A and **(F)** from B

Five weeks after transplantation, the epicenter region in the NSC-only transplanted group was not filled with GFP-labeled NSCs, with the injectate mostly leaking outside of the grafted site (Fig. 5A). In addition, most of the GFP-labeled NSCs showed a spherical morphology (Fig. 5C), and the cells did not grow through the glial scar (Fig. 5E). However, the grafted NSCs in the siRNA-Sema3A-treated group exhibited higher rates of survival in the lesion center than those in the NSC-only transplanted group (Fig. 5B and Fig. S2; SCI + NSC group: 1.00 ± 0.11, SCI + siRNA-Sema3A + NSC group: 2.33 ± 0.15 ^***^p < 0.001). Notably, most of the GFP-labeled NSCs showed an elongated morphology (Fig. 5D), and the cells grew through the glial scar (Fig. 5F). A previous paper reported that the change of NSCs from a spherical morphology to an elongated morphology suggests that differentiation is occurring [29], which indicates that inhibiting Sema3A expression induces neural differentiation and increases the survival of grafted NSCs after transplantation.

#### Knockdown of Sema3A upregulates neuronal differentiation and synaptic contact between host and grafted NSCs

**Fig. 6:**
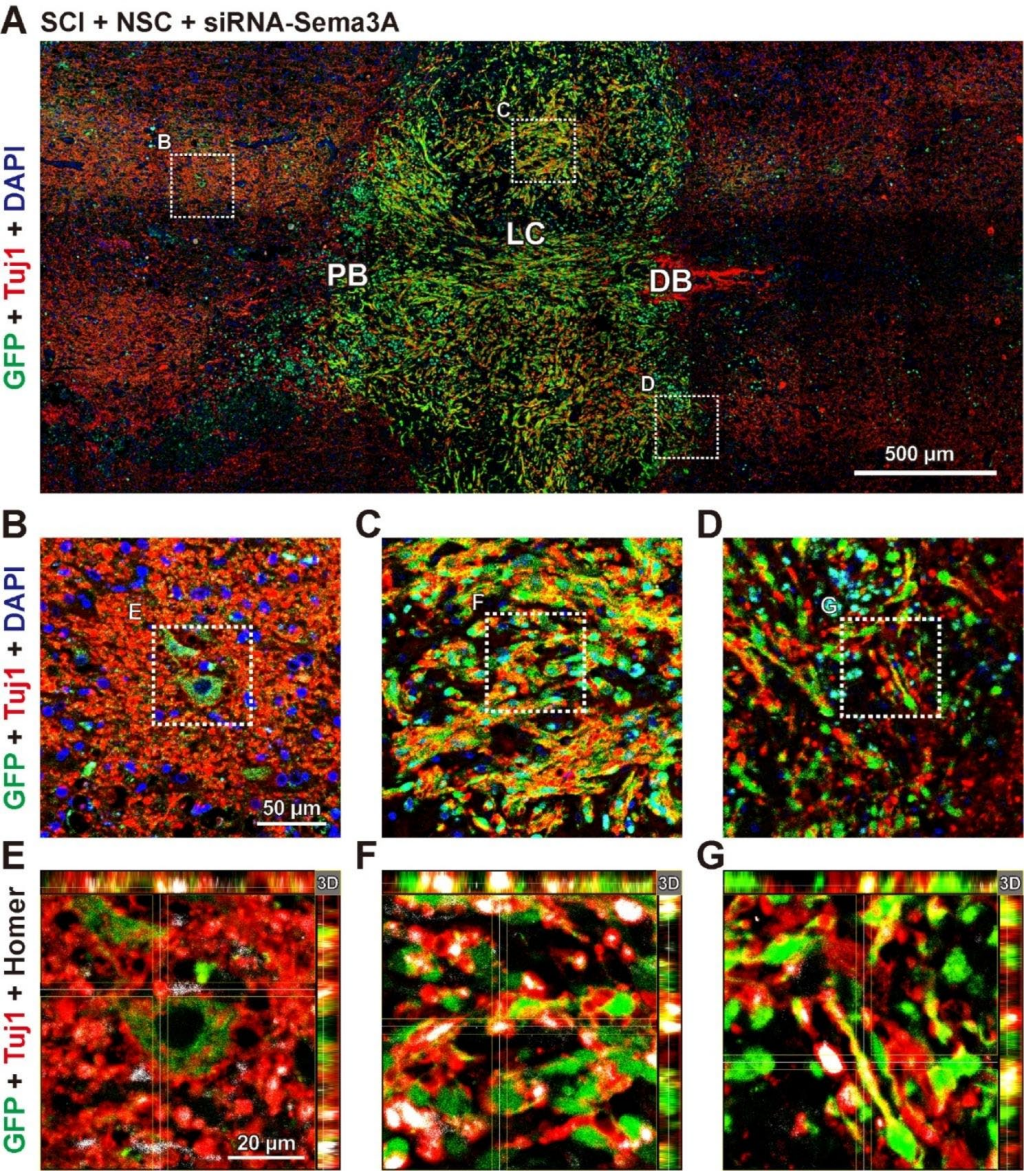
Knockdown of Sema3A can improve synaptic connectivity between grafted NSCs and host neurons after SCI. **(A-G)** Immunofluorescence analysis in merged images of transverse sections stained for GFP-labeled NSCs (anti-GFP, Green), neurons (anti-Tuj1, Red), and nuclei (DAPI, Blue) or synapse (anti-Homer, White). **(A)** Representative composite tiled scan merged images of GFP and GFAP in the SCI + NSC + siRNA-Sema3A group. **(B-D)** Higher magnification for GFP, Tuj1, and DAPI **(B)** ahead of the proximal border (PB), **(C)** at the grafted site, and **(D)** in the distal border (DB) from A. **(E-G)** Higher magnification for GFP, Tuj1, and Homer in the boxed area **(E)** from B, **(F)** from C, and **(G)** from D

To confirm how the knockdown of Sema3A affects the neuronal differentiation of transplanted NSCs, the immunofluorescence with neurons marker, Tuj1, and a postsynaptic marker, Homer, was assessed around the proximal border, lesion center, and distal border in the Sema3A-inhibited rats following NSC transplantation (Fig. 6A). At the proximal border, distal border, and lesion center, GFP-labeled NSCs were localized around Tuj1^+^ host neuronal cells (Fig. 6B, C, D). Most of the grafted GFP-labeled NSCs were colocalized with Tuj1 and Homer markers at the proximal border, lesion center, and distal border (Fig. 6E-G). Notably, the Homer intensity of the siRNA-Sema3A-treated group was more than 11-fold greater than that at the grafted site of the NSC-only transplanted group (Fig. S3; SCI + NSC group: 0.54 ± 0.19, SCI + siRNA-Sema3A + NSC group: 5.95 ± 0.36, ^***^p < 0.001). These data show that synapse formation in transplanted NSCs can be significantly increased by suppressing Sema3A expression after SCI. Overall, this suggests that inhibiting Sema3A expression after NSC transplantation can improve the synaptic connectivity between grafted NSCs and host rat neurons.

### Knockdown of Sema3A enhances laminin expression and motor function recovery after SCI

During embryogenesis and neural development, laminin is an essential extracellular matrix molecule that contributes to biological phenomena such as cell growth, survival, migration, and differentiation [30, 31]. To elucidate the potential mechanisms underlying Sema3A inhibition-mediated cell survival and axon growth, we examined expression rates of the laminin.

**Fig. 7:**
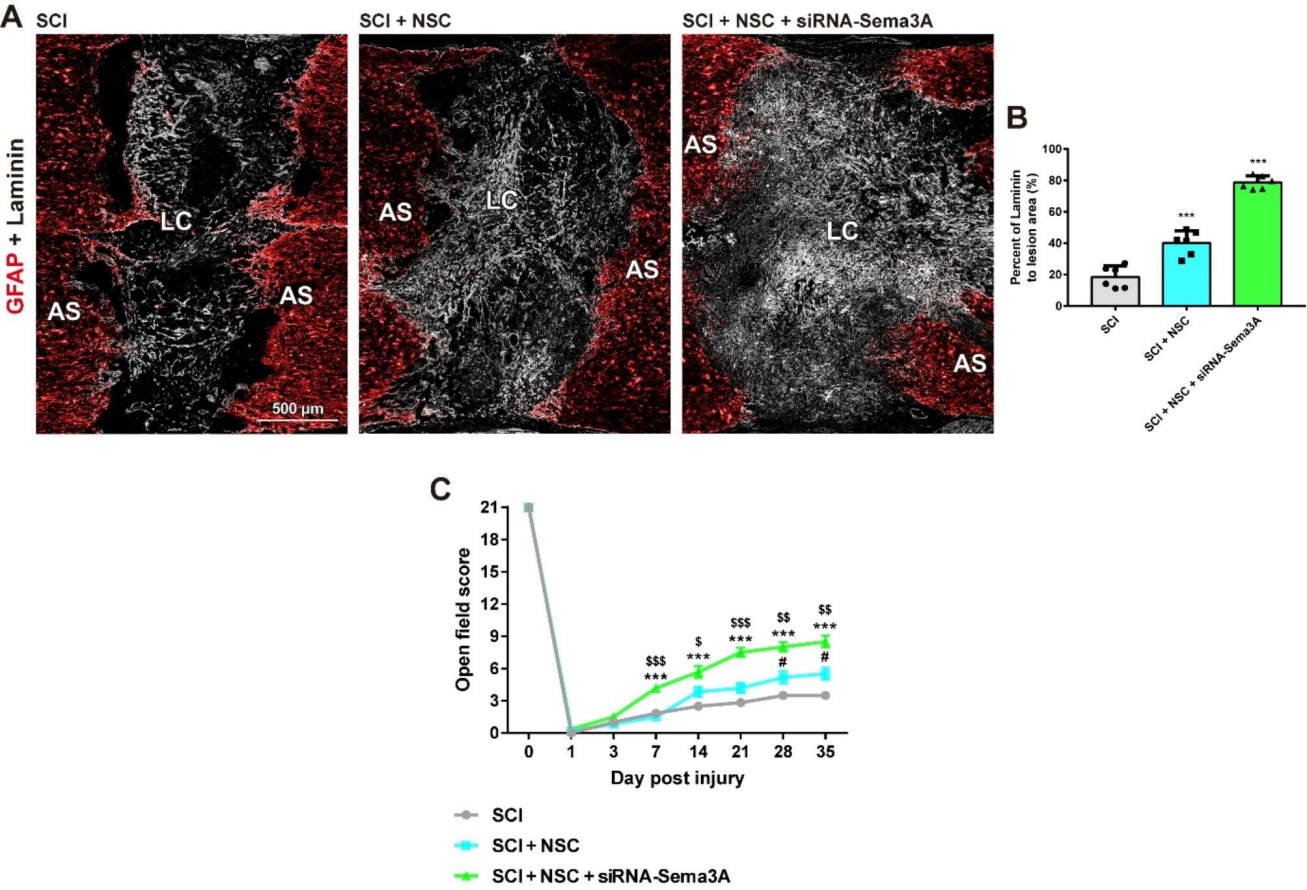
Knockdown of Sema3A enhances laminin expression and improves motor function recovery after SCI **(A)** Immunofluorescence analysis in composite tiled scans of transverse sections stained for AS (anti-GFAP, Red) and laminin (anti-laminin, White). **(B)** Quantitative analyses of the laminin percentage in the LC. Results are the mean ± SEM; ^***^p < 0.001. one-way ANOVA with Tukey post hoc test. **(C)** Comparison of BBB locomotor scores in the SCI, SCI + NSC, and SCI + NSC + siRNA-Sema3A group. ^#^ denotes a significant difference between the SCI group and the SCI + NSC group. ^*^ denotes a significant difference between the SCI group and the SCI + NSC + siRNA-Sema3A group. ^$^ denotes a significant difference between the SCI + NSC group and the SCI + NSC + siRNA-Sema3A group. Results are the mean ± SD; ^#^*p* < 0.05, ^$^*p* < 0.05, ^$$^*p* < 0.01, ^$$$^*p* < 0.001, and ^***^p < 0.001. Unpaired two-tailed Student’s t-tests

Five weeks after SCI, rats exhibited laminin expression intensity rates of 18.53 ± 6.38 in the lesion center (Fig. 7A). However, laminin expression levels after the transplantation of NSCs were significantly increased in the grafted sites compared to those in the SCI rats (Fig. 7B; SCI + NSC group: 40.23 ± 7.17, ^***^p < 0.001). Moreover, laminin expression levels in the grafted sites of the siRNA-Sema3A-treated group following NSC transplantation were significantly increased in the lesion center compared to those in the grafted sites of NSC-only transplanted rats (SCI + siRNA-Sema3A + NSC group: 78.72 ± 3.83, ^***^p < 0.001). Specifically, after the transplantation of NSCs, laminin expression levels in the grafted sites of the siRNA-Sema3A-treated group were more than four-fold greater in the lesion center than those in the injured sites of the SCI group.

To evaluate whether Sema3A suppression could improve functional recovery after SCI, we evaluated the hindlimb locomotor function. After 7 days post injury (DPI), the locomotor scores between the SCI and SCI + NSC groups exhibited no significant differences (Fig. 7C DPI: SCI group: 1.83 ± 0.41, SCI + NSC group: 1.50 ± 0.55), while the locomotor scores of the siRNA-Sema3A-treated group were significantly increased compared to those of the SCI and SCI + NSC groups (7 DPI: SCI + siRNA-Sema3A + NSC group: 4.17 ± 0.75, ^***^ p < 0.001, ^$$$^ p < 0.001). This trend remained consistent until 21 DPI (^***^ p < 0.001, ^$^ p < 0.05). After 28 and 35 DPI, the locomotor scores of the SCI + NSC group were significantly increased compared to those of the SCI group (Figs. 7C and 28 DPI: SCI group: 3.50 ± 0.55, SCI + NSC group: 5.17 ± 1.47, 35 DPI: SCI group: 3.50 ± 0.55, SCI + NSC group: 5.50 ± 1.38, ^#^ p < 0.001). Notably, the locomotor scores of the siRNA-Sema3A-treated group were significantly increased compared to those of the SCI + NSC group (Figs. 7C and 28 DPI: SCI + siRNA-Sema3A + NSC group: 8.00 ± 1.10; 35 DPI: SCI + siRNA-Sema3A + NSC group: 8.50 ± 1.38, ^$$^ p < 0.01). These results suggest that inhibiting Sema3A expression after NSC transplantation can significantly improve function recovery.

## Discussion

The application of NSC transplantation to a CNS injury is highly promising due to the ability of NSCs to replace damaged/lost cells after trauma. Despite the significant progress that has been achieved over decades of research regarding NSC-mediated differentiation and the reformation of neural circuitry [2, 32], their therapeutic efficacy remains insufficient to translate to clinic use [33]. Importantly, the successful reconstruction of the neuronal circuitry can be mediated through increased neuronal differentiation, appropriate axon guidance, and by facilitating synapse formation.

In our recent research, we demonstrated the distinct mechanical properties and degradability of a chitosan-hyaluronic acid hydrogel, which can be controlled by manipulating its constituent ratios [25, 34, 35]. These characteristics can play a dual role, specifically short-term drug release during acute inflammation and the long-term retention of grafted cells at the injured site. Collectively, our findings align with those of other recent studies, showing the potential of a dual-degradable gel with distinct degradation timelines. Taken together, this dual-degradable gel may be clinically applicable as a NSC transplantation therapy following a spinal cord injury.

Sema3A is a chemorepulsive protein that regulates growth cone collapse and it is implicated in axon guidance function, particularly during the development of the CNS [36]. Sema3A is expressed in developing neural cells and adult neurons of the CNS [37–39]; however, its expression in transplanted NSCs has yet to be characterized. Here, we detected Sema3A expression in NSC cultures and transplanted NSCs after SCI. Of note, we observed that NSC-secreted Sema3A demonstrated axon inhibitory guidance capabilities in the form autocrine signaling, which could be reversed by suppressing Sema3A with siRNA. To the best of our knowledge, this is the first study to report that differentiating NSCs can produce Sema3A in vitro and in vivo, which implies that Sema3A suppression can serve as a possible therapeutic mechanism to promote axonal outgrowth after SCI.

Of note, Neuropilin-1 (NRP1) is a major receptor that interacts with Sema3A. VEGF, the major regulator of angiogenesis, is an antagonist that competes with Sema3A for NRP1 binding. Thus, Sema3A acts as a selective inhibitor of VEGF-mediated angiogenesis and it has been shown recently to induce cell apoptosis [40, 41]. We found that the survival of transplanted NSCs in Sema3A-inhibited group was significantly increased compared with that in NSC-only transplanted rats after SCI. This indicates that NSC survival may be due to the increased VEGF binding with NRP1.

Recently, Kaneko et al. reported that Sema3A plays a guidance role during axonal regeneration and identified an enhanced regenerative response and functional recovery by suppressing Sema3A after SCI [42]. Other studies have shown that interference with Sema3A is beneficial for synaptogenesis and functional recovery following CNS trauma [43–45]. In this study, we corroborated previous findings, as our results suggest that Sema3A suppression significantly promotes synaptic formation and the behavioral recovery of Sema3A-inhibited rats following NSC transplantation compared to those in NSC-only transplanted rats after SCI. Importantly, we confirmed that Sema3A expression is increased following NSC transplantation, localizing at the grafted site.

## Conclusions

Our findings show that Sema3A is expressed in differentiating NSCs and that this can be suppressed to increase the survival of transplanted NSCs. Subsequently, the knockdown of Sema3A can promote neuronal differentiation and thereby synapse formation between host neurons and transplanted cells. Therefore, we suggest that suppressing Sema3A should be an essential consideration in future cell transplantation therapies for SCI.

### Electronic supplementary material

Below is the link to the electronic supplementary material.

Supplementary Material 1

## Data Availability

All relevant data are available within the article and its supplementary information files, or available from the corresponding authors upon reasonable request.

## Abbreviations

SCI: Spinal cord injury
CNS: Central nervous system
NSC: Neural stem cell
Sema3A: Semaphorin-3 A
AuNP@siRNA-Sema3A: Sema3A siRNA-loaded gold nanoparticles
AuNPs: Gold nanoparticles
DW: Deionized water
siRNA-Sema3A: siRNA of Sema3A
PLL: Poly-l-lysine
GFP: Green fluorescent protein
DAPI: 4′6′-diamidino-2-phenylindole dihydrochloride
Cy5.5: Cyanine 5.5 dye
ROI: Regions of interest
gC: Glycol chitosan
oHA: Oxidized hyaluronic acid
IACUC: Institutional animal care and use committee
VEGF: Vascular epidermal growth factor
BBB: Basso, Beattie and Bresnahan
ANOVA: Analysis of variance
DPI: Days post injury
NRP-1: Neuropilin-1
