# Supplementary material for: Axon guidance gene-targeted siRNA delivery system improves neural stem cell transplantation therapy after spinal cord injury
Source: Biomater Res. 2023 Oct 15;27:101. doi: 10.1186/s40824-023-00434-2 (PMC10577901; doi:10.1186/s40824-023-00434-2)
Supplement: Supplementary file 1 — Supplementary Material 1 [file 40824_2023_434_MOESM1_ESM.docx]

Supplementary material for

**Axon guidance gene-targeted siRNA delivery system improves neural stem cell transplantation therapy after spinal cord injury**

Seong Jun Kim ^1,2^, Wan-Kyu Ko ^1,2^, Gong Ho Han ^1,2^, Daye Lee ^1,2^, Min Jai Cho ^3^, Seung Hun Sheen ^1^, and Seil Sohn ^1,2*^

^1^ Department of Neurosurgery, CHA Bundang Medical Center, CHA University; 59, Yatap-ro, Bundang-gu, Seongnam-si, Gyeonggi-do, 13496, Republic of Korea

^2^ Department of Biomedical Science, CHA University; 335, Pangyo-ro, Bundang-gu, Seongnam-si, Gyeonggi-do, 13488, Republic of Korea

^3^ Department of Neurosurgery, Chungbuk National University; 776, 1Sunhawn-ro, Seowon-gu, Cheongju-si, 28644, Republic of Korea

^*^Seil Sohn, MD, PhD

Department of Neurosurgery,

CHA University College of Medicine

59, Yatap-ro, Bundang-gu, Seongnam-si, Gyeonggi-do, 13496, Korea

Phone: 82-31-881-7966

Fax: 82-2-780-5269

E-mail: sisohn@cha.ac.kr

^*^ Seil Sohn is the corresponding author.


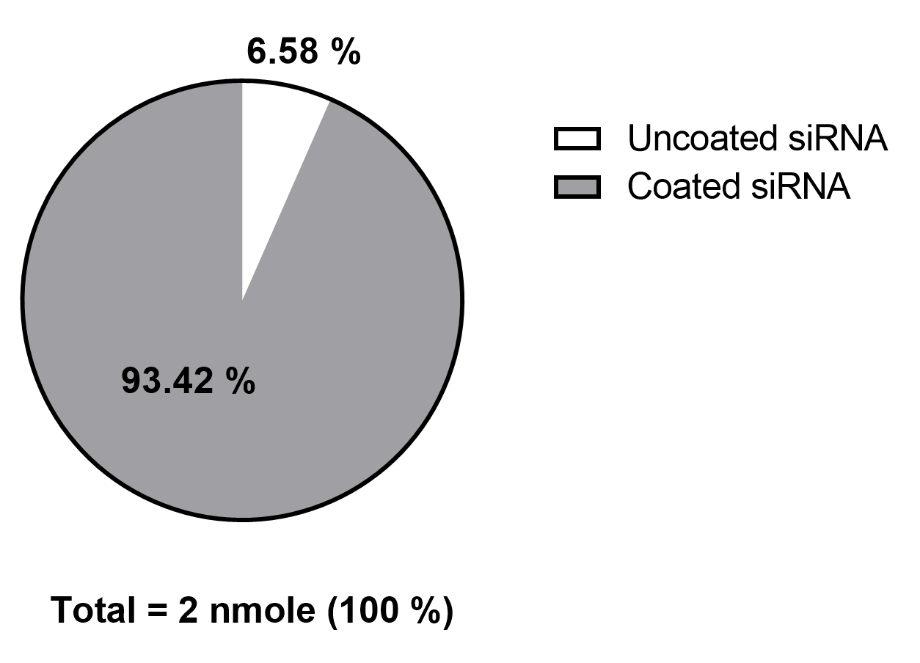


**Fig. S1** Coating ratio of siRNA-Sema3A with AuNPs


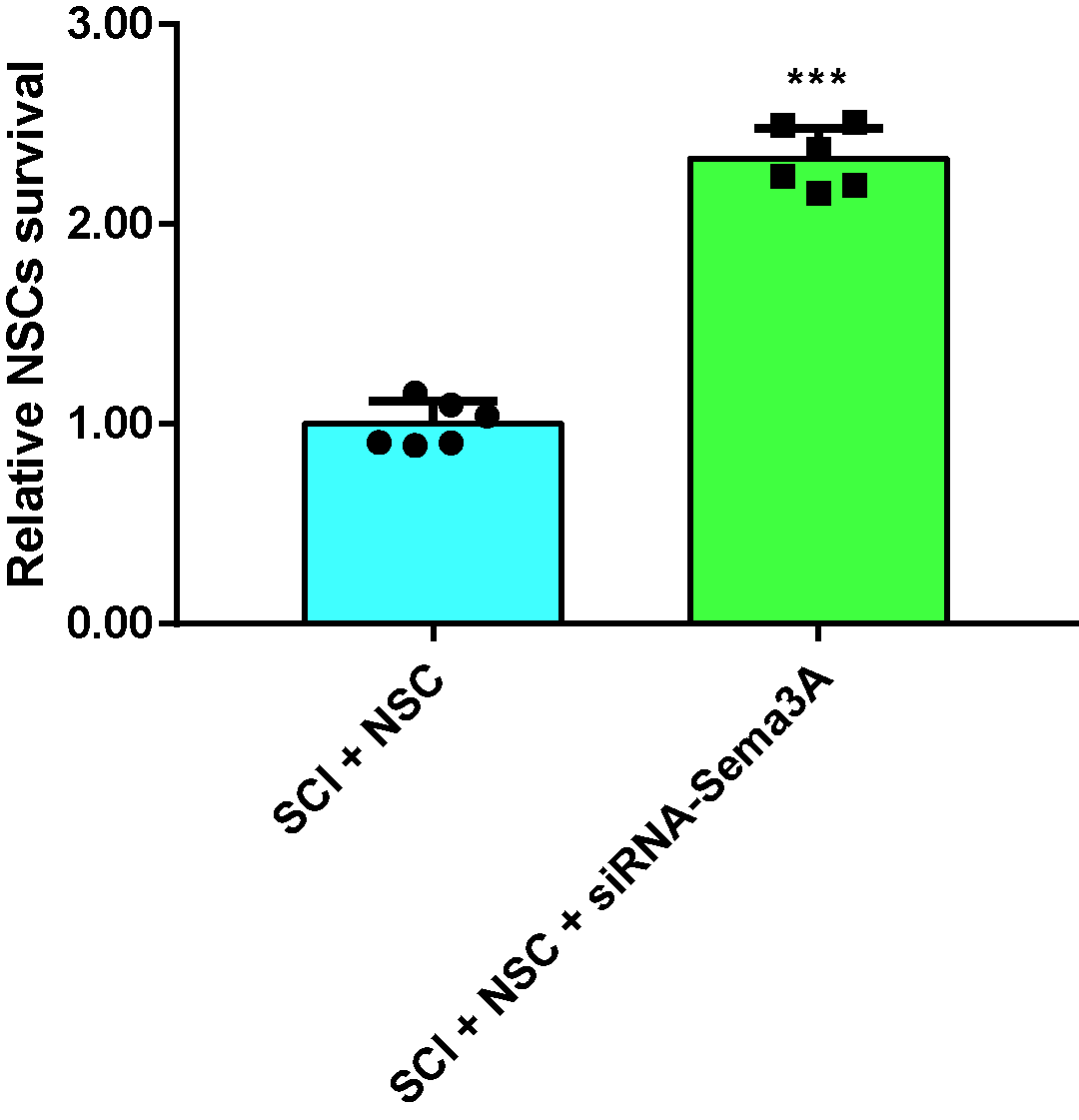


**Fig. S2** Knockdown of Sema3A increases the survival of grafted NSCs after SCI**.** Quantitative analyses of the GFP intensity in the LC. Results are the mean ± SEM; ^***^ *p* < 0.001. one-way ANOVA with Tukey post-hoc test.


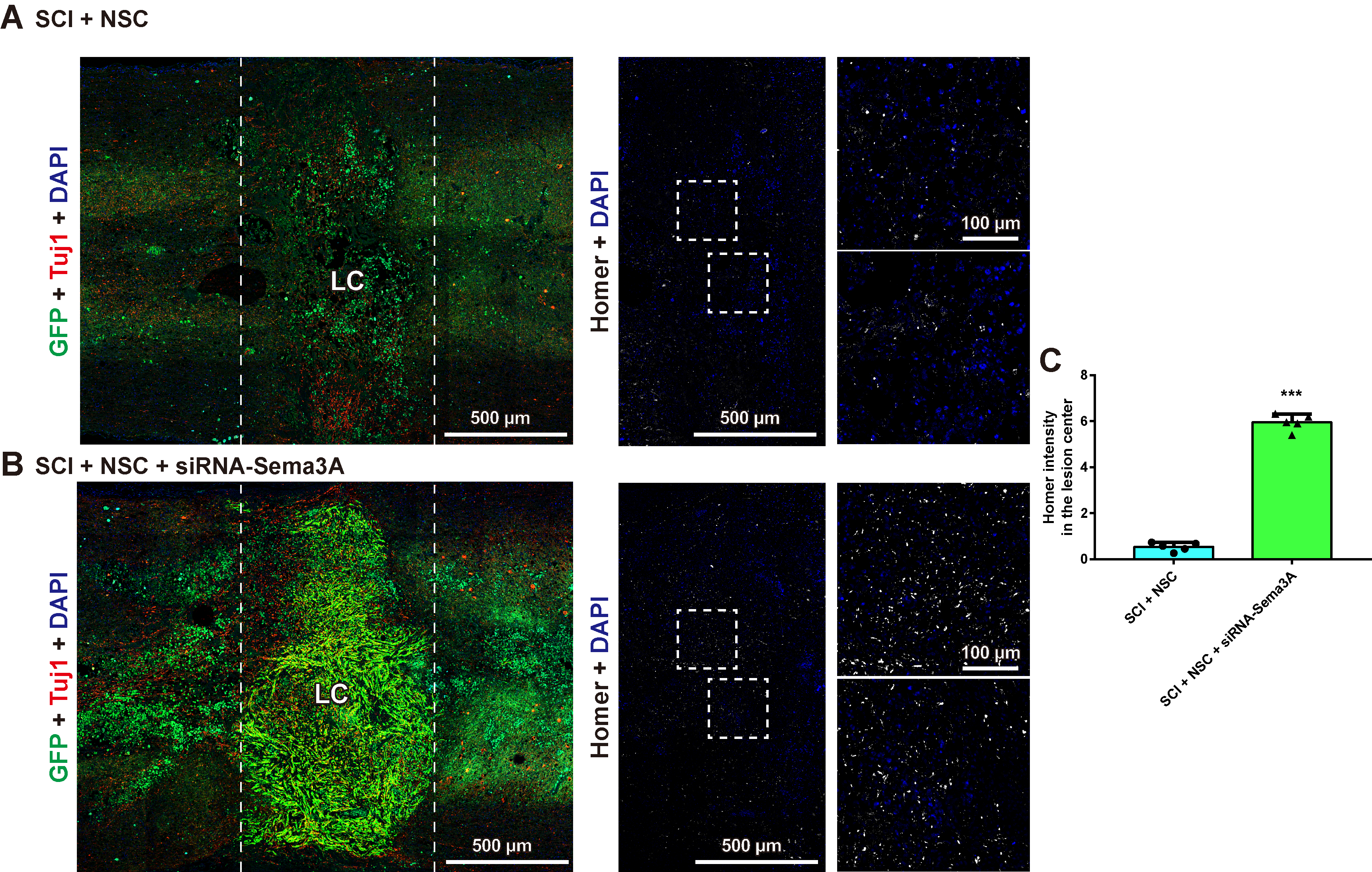


**Fig. S3** Knockdown of Sema3A in transplanted NSCs can improve synaptic connectivity between grafted NSCs and host neurons after SCI. **(A)** Immunofluorescence analysis in composite tiled scans of transverse sections stained for GFP-labeled NSCs (anti-GFP, Green), neurons (anti-Tuj1, Red), and nuclei (DAPI, Blue) in the SCI + NSC group (left). Higher magnification for the synapse (anti-Homer, White) and DAPI in the lesion center (LC; right) **(B)** Immunofluorescence analysis in composite tiled scans of transverse sections stained for GFP-labeled NSCs (anti-GFP, Green), neurons (anti-Tuj1, Red), and nuclei (DAPI, Blue) in the SCI + NSC + siRNA-Sema3A group (left). Higher magnification for the synapse (anti-Homer, White) and DAPI in the LC (right). **(C)** Quantitative analyses of the Homer intensity in the LC. Results are the mean ± SEM; ^***^ *p* < 0.001. one-way ANOVA with Tukey post-hoc test.
